# The effect of virtual reality technology on the intervention of mental health problems among healthcare workers: a systematic review

**DOI:** 10.3389/fpubh.2026.1735214

**Published:** 2026-04-09

**Authors:** Tian Chen, Caixia Xie, Xiaoxia Dai

**Affiliations:** 1 School of Medicine, University of Electronic Science and Technology of China, Chengdu, China; 2 Department of Nursing, Sichuan Provincial People’s Hospital, University of Electronic Science and Technology of China, Chengdu, China

**Keywords:** anxiety, mental health problem, nurse, physician, stress, virtual reality technology

## Abstract

**Purpose:**

Systematic evaluation of the effectiveness of virtual reality technology in intervening in mental health problems of healthcare workers (PROSPERO: CRD42024558009).

**Methods:**

PubMed, The Cochrane Library, Embase, Web of Science, CNKI, China Wanfang Database, and China Biology Medicine disk were systematically searched from inception to May 21, 2025. We included experimental and quasi-experimental studies that examined the effects of VR interventions on mental health outcomes (stress, anxiety, burnout, or fatigue) in healthcare workers. Studies were excluded if they were not published in English or Chinese, if full texts or required data were unavailable, or if they were duplicate publications. The review followed the Preferred Reporting Items for Systematic Reviews and Meta-Analyses (PRISMA) guidelines. Methodological quality was assessed using the Effective Public Health Practice Project (EPHPP) tool, and meta-analyses were performed using RevMan 5.4 software.

**Results:**

2,133 articles were identified through database searching. Ten studies published between 2021 and 2023, with 493 participants, were included in the review. Of these, there were three randomized cross-over trials, and seven were one-group pretest-posttest trials. The findings showed that both the stress and anxiety scores before and after the VR intervention were statistically significant [SMD = −0.64, 95% CI (−1.17, −0.11), *p* = 0.02], and [SMD = −0.51, 95% CI (−0.76, −0.27), *p* < 0.0001]. Since a meta-analysis of the burnout, fatigue, and satisfaction levels was not possible, a descriptive analysis was carried out. Exhaustion and burnout may be lessened following the intervention. The majority of the medical team expressed satisfaction with VR’s ability to alleviate mental health issues. Three studies received an EPHPP rating of ‘strong’, five were ‘moderate’, and two were ‘weak’.

**Conclusion:**

Existing evidence suggests that virtual reality technology is effective in improving stress and anxiety in healthcare workers, but more high-quality studies are needed to further validate whether it can improve burnout and fatigue.

## 1 Introduction

According to the World Health Organization (WHO), mental health is a state of well-being that enables individuals to cope with life stressors, realize their abilities, learn and work effectively, and contribute to their communities (1). Common mental health challenges include stress, anxiety, burnout, and fatigue. Healthcare workers are particularly vulnerable to psychological distress due to high work pressure, exposure to stressful situations, complex interpersonal interactions, and demanding patient care responsibilities (2). The prevalence of these mental health problems among healthcare professionals has reached concerning levels, with studies reporting rates of 18.19% for stress (3), 31.9% for anxiety (4), 40.4% for burnout (5), and between 27 and 80% for fatigue (6). Given that healthcare workers constitute the core of health systems, their mental health status directly impacts patient safety, service quality, and overall system efficiency (7).

Innovative approaches to addressing these mental health challenges are urgently needed. Virtual reality (VR) technology, characterized by its immersive, interactive, and computer-generated environments, has emerged as a promising tool for mental health interventions (8, 9). VR offers unique advantages, including controlled exposure to stressful scenarios, immersive relaxation experiences, and the ability to deliver interventions in accessible, standardized formats. In recent years, VR has been increasingly employed for mental health treatment in patient populations (10). However, the application of VR technology specifically for healthcare workers’ mental health remains relatively underexplored. While several systematic reviews have examined VR interventions for mental health in general populations or specific patient groups (11, 12), no comprehensive synthesis has focused specifically on healthcare workers—a population facing unique occupational stressors and barriers to seeking traditional mental health support. The existing primary studies examining VR interventions for healthcare workers have yielded variable findings, and the overall effectiveness remains unclear due to differences in intervention protocols, outcome measures, and study designs.

Therefore, this systematic review and meta-analysis aims to: (a) synthesize available evidence on the effectiveness of VR interventions in improving mental health outcomes (stress, anxiety, burnout, fatigue, and satisfaction) among healthcare workers; (b) quantify the overall effect sizes for outcomes with sufficient data; and (c) identify knowledge gaps to inform future research directions. By providing a comprehensive evidence synthesis, this review seeks to establish an evidence-based foundation for implementing VR interventions to support healthcare workers’ mental well-being and to guide the development of targeted digital mental health strategies for this critical workforce(PROSPERO: CRD42024558009).

## 2 Methods

### 2.1 Search strategy

We first confirmed that no similar review existed in the Cochrane Library and PROSPERO. This review was carried out by the Preferred Reporting Items for Systematic Reviews and Meta-Analyses (PRISMA) (13). PubMed, The Cochrane Library, Embase, Web of Science, CNKI, China Wanfang database, and the China Biology Medicine disk were searched until May 21, 2025. Search Medical Subject Headings (MESH) were: “Virtual Reality” AND “Health Personnel” OR “Physicians” OR “Nurses” OR “Anesthesiologists” AND “Mental Health” OR “Stress, Psychological” OR “Anxiety” OR “Burnout, Professional” OR “fatigue.” A combination of Medical Subject Headings (MESH) and free-text terms was used in our search strategy. Studies were extracted and screened using the reference management software Endnote. All stages of the search strategy and data extraction were carried out by two independent reviewers. Any discrepancy was resolved by discussion with a third reviewer. There was regular checking and consultation on this process between the research team to ensure that relevant articles were not overlooked.

### 2.2 Inclusion and exclusion criteria

Studies were included in the review if they met the following criteria: (a) Type of study: experiment studies, quasi-experiment studies; (b) Population: healthcare workers engaged in clinical frontlines; (c) Intervention: interventions involving VR technology without limiting the specific type of VR; (d) Outcome: mental health problems such as stress, anxiety, burnout, and fatigue as well as satisfaction. Papers were excluded if they were (a) published in a language other than English or Chinese; (b) full text or required data were inaccessible; or (c) duplicate publications.

### 2.3 Quality assessment

Quality ratings of included studies were carried out by two independent reviewers (TC and XD) trained in evidence-based nursing knowledge using the Effective Public Health Practice Project (EPHPP) quality assessment tool for quantitative studies (14). Any discrepancies were resolved through a discussion with a third reviewer (CX). EPHPP can provide consistent quality ratings for a range of study designs. EPHPP rates six methodological domains: selection bias, study design, confounders, blinding, data collection method, and withdrawals and Dropout. For every study, an overall rating is determined. If there are no weak subscale ratings, studies are rated as “strong” overall, “moderate” if only one subscale grade is weak, and “weak” if two or more ratings on the weak subscale are present.

### 2.4 Strategy for data synthesis

Meta-analysis was performed using RevMan5.4 software. The measurement data adopts standardized mean difference (SMD) and 95% confidence interval (CI) as effect indicators, with *p* < 0.05 indicating statistical significance. Use the χ^2^ test to determine whether there is heterogeneity among the studies. If *p* ≥ 0.1 and I^2^ ≤ 50%, it indicates small heterogeneity and a fixed effects model is chosen. On the contrary, it indicates significant heterogeneity and requires subgroup or sensitivity analysis to identify the source of heterogeneity. A random effects model is chosen, if the source of heterogeneity cannot be explained by clinical and methodological heterogeneity.

## 3 Results

### 3.1 Study characteristics

**Figure 1:**
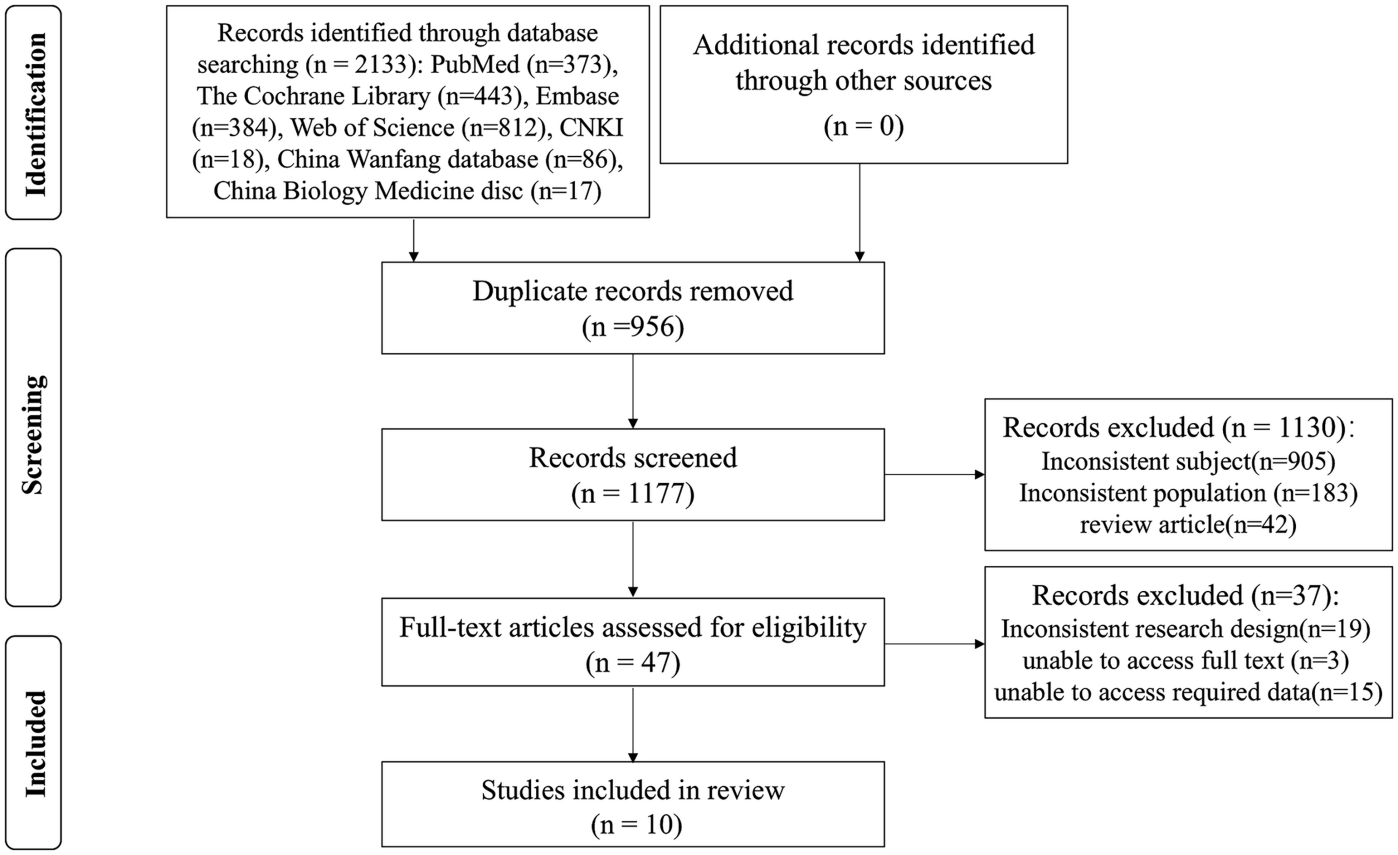
Preferred reporting items for systematic reviews and meta-analyses (PRISMA) flow diagram of studies investigating virtual reality technology for healthcare workers with mental health problems.

A total of 2,133 articles were identified through database searching. The full texts of 47 papers were screened, ten met the inclusion criteria and were included in the review. See Figure 1 for the Preferred Reporting Items for Systematic Reviews and Meta-Analyses (PRISMA) flow diagram. See Table 1 for study characteristics. The 10 included studies were published between 2021 and 2023. There were three randomized cross-over trials (15–17), while seven were one-group pretest-posttest trials (18–24). Specifically, studies were conducted in France (*N* = 1), The United States of America (*N* = 4), The Netherlands (*N* = 1), China (*N* = 1), Italy (*N* = 1), Japan (*N* = 1), and Spain (*N* = 1). A total of 493 participants took part in the studies. The number of participants recruited across the studies ranged from 4 to 102. Five studies had samples of over 50 participants. Six studies used health personnel participants. Three studies used nurse participants. One study used physician participants. The duration of each session ranged from 3 min to half an hour.

**Table 1:** Characteristics of studies of virtual reality technology for healthcare workers with mental health problems.

Study	Country	Study design	Participants	*N*	Intervention	Measures	Findings
Bodet-Contentin et al. (15)	France	Randomized cross-over trial	Intensive unit caregiver	88	Each caregiver will have two shifts: one with a VR session during the breaktime and one without a VR session. The VR session was 8 min long and implemented using the Healthy Mind VR program and an Oculus GO VR device with an audio headset. Three natural virtual environments were available: a garden, forest, or mountain.	VAS-F; VAS-S; VAS-A; satisfaction	VR induced a significantly higher reduction in the fatigue score. 68% of participants were satisfied with the VR session. No significant difference was observed for other parameters.
Weitzman et al. (16)	USA	Randomized cross-over trial	Otolaryngology Resident	18	The first group was in the intervention phase. They were assigned 10 min of weekly VR-guided meditation using a smartphone app that guided users through paced breathing and mindfulness meditation exercises in the backdrop of three virtual environments: beach, canyon, or forest. The software combined a smartphone fitted to a Samsung Gear Oculus VR headset. The second group did not undergo the intervention during this time.	MBI; satisfaction	Weekly VR-guided meditation and paced breathing were associated with a significant decrease in emotional exhaustion. 74% of participants were satisfied with the VR-guided meditation.
Nijland et al. (18)	The Netherlands	One-group pretest-posttest trial	Intensive Care Nurse	66	The nurses were encouraged to use VRelax as a short break during their shift. The device was an Oculus Go stand-alone head-mounted display running the VRelax application. The participants could navigate through high-quality immersive 360-degree videos of calming natural environments. Options include walking on a beach and underwater swimming with wild dolphins. The recommended minimal time of use was 10 min; a longer duration was allowed.	VAS-S	Statistically significant difference of the mean VAS stress before and after use.
Beverly et al. (19)	USA	One-group pretest-posttest trial	Healthcare worker	102	Participants volunteered to view an immersive three-minute 360-degree Cine-VR scene of a local nature preserve via an Oculus Go or Pico G2 4K HMDs. Cine-VR leverages 360-video techniques with cinema techniques to create VR.	VAS-S	A significant reduction in subjective stress scores from pre- to post-simulation
Croghan et al. (17)	USA	Randomized cross-over trial	Health Care Professional	24	The intervention consisted of viewing 2 nature-based scenes (“walk in the woods” and “forest of focus”) through Oculus VR goggles and with computer 4 K graphic imagery. The viewing order was randomly assigned. Study participants were scheduled during the workday for four 30-min study visits within a 2 to 3-week time period to view the nature-based intervention. These visits needed to have at least one day in-between viewings.	STAI	All 4 sessions had a significant decrease in score from pre to post, but there was no significant difference in the change from pre- to post-session between the 4 groups.
Pan et al. (20)	China	One-group pretest-posttest trial	Health Care Professional	4	The setting of therapy was designed to receive 30 min of VRT psychological intervention at a specific location, every day for a week, in total seven times. The hardware used a China-made integrated VR machine with a binocular resolution of 2.5 K. Software was developed on 3D Unity, which contained 12 different virtual scenarios, including grassland, seabed, beach, forest, etc.	GAD-7	Cases showed a reduction in scale comparison. General scores of GAD-7 reduced 52.17%. Meanwhile, a reduction in anxiety symptoms was also found.
Pallavicini et al. (21)	Italy	One-group pretest-posttest trial	Healthcare worker	20	MIND-VR is an immersive VR psychoeducational experience on stress and anxiety that includes an HMD and two controllers. We chose a small tropical island as the setting. On the virtual island, three paths describe different aspects related to stress and anxiety: (1) definitions (first path); (2) causes and symptoms (second path); and (3) main treatments (third path). The completion of each route takes about 15 min.	STAI; satisfaction	The virtual reality psychoeducational experience decreased state anxiety and was highly usable and satisfying.
Matsumoto et al. (22)	Japan	One-group pretest-posttest trial	Nurse	65	Experience the VR app (10 min) (The goal of the current investigation, which was a randomized controlled experiment, was to confirm that using chatbots and virtual reality together lessened emotional stress than using either tool alone. Prior to the randomized controlled study, individuals participated in a self-pre- and post-control trial of the VR experience to evaluate the technology’s ability to reduce stress. As a result, the data from this portion of the trial were chosen for analysis.)	STAI	There was a significant improvement in the change of scores.
Hayakawa et al. (23)	USA	One-group pretest-posttest trial	Pediatric Healthcare Worker	71	The system is a deeply immersive, gesture-controlled VR platform that merges music, movement, and visuals to provide players with moments of delight, enabling an emotionally compelling and healing therapeutic experience. This study used Two types of VR headsets: the Oculus Rift and the VIVE Focus. The intervention was 3 min 40 s each time for a total of 3 sessions.	STAI	STAI anxiety scores were significantly decreased after VR intervention.
Linares-Chamorro et al. (24)	Spain	One-group pretest-posttest trial	Health Professional	35	The intervention was a VR experience using a projection device with light and sound control. Each intervention lasted 10 min, twice a week for 8 weeks.	HAMA	A statistically significant improvement in anxiety was found.

KEY: VAS-F visual analog scale for fatigue, VAS-S visual analog scale for stress, VAS-A visual analog scale for anxiety, MBI Maslach Burnout Inventory, STAI State–trait anxiety inventory, GAD-7 General Anxiety Disorder-7, HAMA Hamilton Anxiety Scale.

### 3.2 Stress

**Figure 2:**
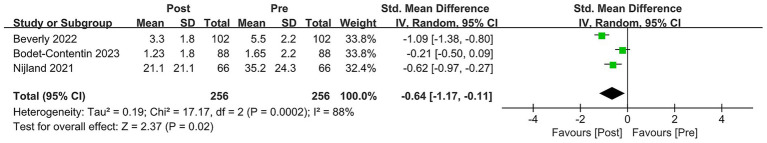
Meta-analysis of the effects of VR interventions on stress in healthcare workers.

Three studies (15, 18, 19) evaluated the effect of VR on stress in healthcare workers. The studies were heterogeneous (*p* = 0.0002, *I*^2^ = 88%), and using a random-effects model, the results demonstrated that the differences before and after the VR intervention were statistically significant [SMD = −0.64, 95% CI (−1.17, −0.11), *p* = 0.02]. The study effect sizes did not change significantly after the literature was excluded one by one for sensitivity analysis, and the results were more robust. See Figure 2.

### 3.3 Anxiety

**Figure 3:**
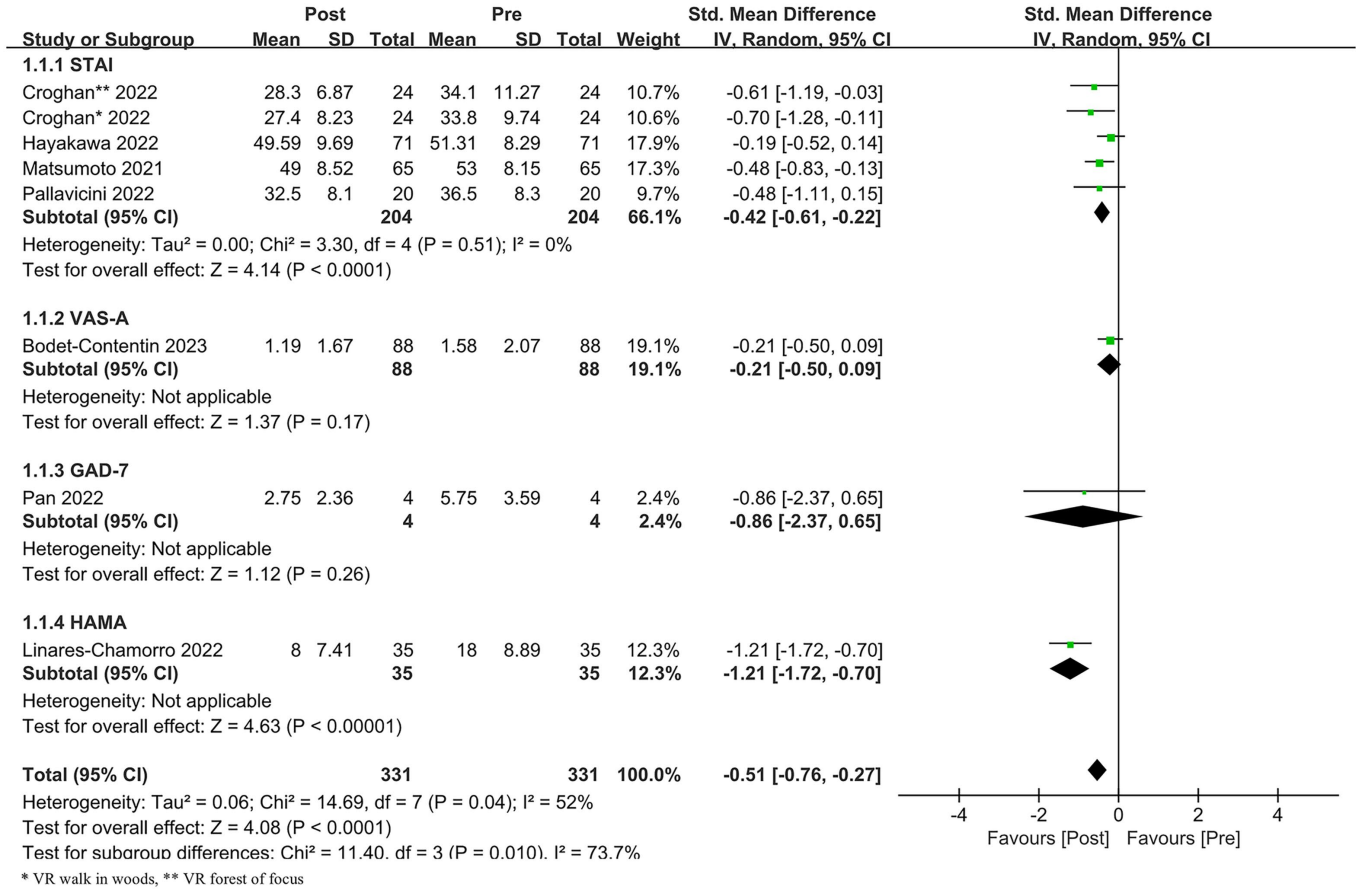
Meta-analysis of the effects of VR interventions on anxiety in healthcare workers.

Seven studies (15, 17, 20–24) evaluated the effect of VR on anxiety in healthcare workers. There was heterogeneity among the studies (*p* = 0.04, *I*^2^ = 52%). Using the random effects model and subgroup analysis based on the evaluation tool, there were four studies with STAI (*p* = 0.51, *I*^2^ = 0%). After excluding the literature one by one for the sensitivity analysis, there was no significant change in the effect value of the studies, and the results were more robust. The results showed that the difference between pre- and post-VR interventions was statistically significant [SMD = −0.51, 95% CI (−0.76, −0.27), *p* < 0.0001]. See Figure 3.

### 3.4 Burnout and fatigue

One study (16) evaluated the effect of VR on burnout in healthcare workers. Thus meta-analysis could not be performed, and its findings showed statistically significant scores on the emotional exhaustion subscale of burnout before and after the VR intervention (*p* = 0.009) and statistically insignificant scores on the depersonalization and personal accomplishment subscales. Another study (15) assessed the effect of VR on healthcare worker fatigue, so meta-analysis could not be performed, and its findings showed statistically significant differences before and after the VR intervention (*p* < 0.01), but the effect was short-lived.

### 3.5 Satisfaction

Three studies (15, 16, 21) reported satisfaction with VR, but they were assessed in different ways, and data were incomplete, so meta-analysis could not be performed. Their findings showed that most healthcare professionals were satisfied with VR to alleviate mental health problems, of which two papers (15, 16) assessed their satisfaction by self-administered questionnaires at approximately 68 and 74%, respectively, and one paper (21) used a Net Promoter Score (NPS) questionnaire to measure their satisfaction. Their mean score was 87, with a standard deviation of 15.5.

### 3.6 Quality ratings

Three studies were given a global EPHPP rating of ‘strong’, five were rated ‘moderate’, and two were rated ‘weak’. See Table 2 for full-quality ratings. Seven of the 10 studies were moderate for selection bias, as they were judged to be somewhat likely to be representative of the target population. Three of the 10 studies were rated as vital for study design because participants were randomly allocated to conditions. The other seven items were a one-group pretest-posttest trial and a lack of control group. Seven of the 10 studies were rated as weak for their control of confounding variables, as little or no information was provided. All studies were rated as having moderate blinding. Because no information was supplied regarding whether the participants knew the research questions or whether the outcome assessor knew about the intervention. For data collection methods, seven out of 10 studies were rated strong. Seven of the 10 studies were rated strong for withdrawals and dropouts, as 80–100% of their participants completed the study.

**Table 2:** Quality assessment using effective public health practice project of studies of virtual reality technology for healthcare workers with mental health problems.

Study	A. Selection bias	B. Study design	C. Confounders	D. Blinding	E. Data collection method	F. Withdrawals and drop out	Global rating
Bodet-Contentin et al. (15)	Moderate	Strong	Moderate	Moderate	Strong	Moderate	Strong
Weitzman et al. (16)	Moderate	Strong	Strong	Moderate	Strong	Moderate	Strong
Nijland et al. (18)	Moderate	Moderate	Weak	Moderate	Strong	Weak	Weak
Beverly et al. (19)	Strong	Moderate	Weak	Moderate	Moderate	Strong	Moderate
Croghan et al. (17)	Moderate	Strong	Strong	Moderate	Strong	Strong	Strong
Pan et al. (20)	Moderate	Weak	Weak	Moderate	Moderate	Strong	Weak
Pallavicini et al. (21)	Strong	Moderate	Weak	Moderate	Strong	Strong	Moderate
Matsumoto et al. (22)	Strong	Moderate	Weak	Moderate	Strong	Strong	Moderate
Hayakawa et al. (23)	Moderate	Moderate	Weak	Moderate	Moderate	Strong	Moderate
Linares-Chamorro et al. (24)	Moderate	Moderate	Weak	Moderate	Strong	Strong	Moderate
Total	3 Strong; 7 Moderate; 0 Weak	3 Strong; 6 Moderate; 1 Weak	2 Strong; 1 Moderate; 7 Weak	0 Strong; 10 Moderate; 0 Weak	7 Strong; 3 Moderate; 0 Weak	7 Strong; 2 Moderate; 1 Weak	3 Strong; 5 Moderate; 2 Weak

## 4 Discussion

### 4.1 Stress

The pooled effect size for stress (SMD = −0.64) indicates a moderate to large effect according to Cohen’s conventions, suggesting that VR interventions can meaningfully reduce perceived stress among healthcare workers. This finding is consistent with previous research (25) and may be explained by several psychological mechanisms. First, VR provides an immersive, stress-relieving environment that allows healthcare workers to temporarily disengage from demanding clinical settings and enter calming virtual scenarios (8). This process aligns with Attention Restoration Theory, which posits that exposure to restorative environments can replenish directed attention capacity depleted by prolonged effort. Second, VR interventions often incorporate relaxation techniques such as guided breathing or progressive muscle relaxation within engaging virtual contexts, potentially enhancing adherence and engagement compared to traditional relaxation methods (26). Third, the sense of presence unique to VR may facilitate deeper emotional and physiological relaxation by creating the illusion of actually “being” in a peaceful environment (9).

Despite the significant pooled effect, the high heterogeneity (I^2^ = 88%) observed in the stress meta-analysis warrants careful interpretation. This substantial statistical heterogeneity suggests that true intervention effects vary considerably across studies and reduces confidence in the precise magnitude of the pooled estimate. Through detailed examination of included studies, we identified multiple potential sources of heterogeneity: First, intervention characteristics varied substantially across studies. Second, study design heterogeneity contributed to variability. Third, participant characteristics differed across studies, including variations in healthcare worker populations, clinical settings, and baseline symptom severity. Sensitivity analyses demonstrated that the pooled effect size remained relatively stable after sequential removal of individual studies, suggesting that no single study disproportionately influenced the overall estimate. However, the high heterogeneity indicates that subgroup analyses or meta-regression would be valuable to explore effect modifiers, though the limited number of studies precluded such analyses. Future research with larger samples and standardized protocols should investigate whether specific intervention characteristics or participant factors moderate treatment effects.

### 4.2 Anxiety

The pooled effect size for anxiety (SMD = −0.51) represents a moderate effect, indicating that VR interventions can significantly reduce anxiety symptoms among healthcare workers. This finding aligns with previous research on VR applications for anxiety in general populations (27, 28) and extends evidence specifically to healthcare worker populations. Anxiety is a prevalent and debilitating condition in healthcare settings, affecting cognitive function, decision-making, and overall well-being (6, 7). VR may ameliorate anxiety through several mechanisms: providing safe, controllable environments for emotional regulation (9); facilitating relaxation responses through immersive calming scenarios; and potentially serving as a distraction from anxiety-provoking thoughts or workplace stressors.

Subgroup analysis based on assessment tools revealed that the effect remained significant for studies using the State–Trait Anxiety Inventory (STAI), the most commonly employed measure. However, the limited number of studies using other assessment tools precluded meaningful subgroup comparisons. The moderate heterogeneity (*I*^2^ = 57%) observed for anxiety outcomes, while lower than for stress, still indicates meaningful variability across studies that likely reflects similar sources of clinical and methodological diversity discussed for stress outcomes.

### 4.3 Burnout and fatigue

Notably, despite burnout being identified as one of the most prevalent mental health challenges among healthcare workers (prevalence up to 40.4% (5)), only one included study (16) examined burnout outcomes. This finding underscores a critical gap in the literature and highlights the need for future VR intervention trials to incorporate burnout as a primary outcome measure. Weitzman et al. (16) reported improvements in emotional exhaustion—often considered the core dimension of burnout—but no significant changes in depersonalization or personal accomplishment. This pattern may reflect the sequential development of burnout, where emotional exhaustion emerges first and may be more responsive to immediate stress-reduction interventions, while depersonalization and reduced personal accomplishment develop more gradually and may require longer-term or multi-component interventions (29). This interpretation aligns with Hatta et al. (28), who similarly found differential effects across burnout dimensions.

For fatigue, the single available study (15) reported transient benefits that did not persist beyond the immediate intervention period. This finding may reflect the multifactorial nature of fatigue, which encompasses physical, mental, and emotional components influenced by workplace factors, individual factors, and organizational factors (30). As Bodet-Contentin et al. (13) noted, addressing fatigue solely at the individual level through brief VR interventions may be insufficient; organizational-level interventions addressing scheduling, workload, and recovery opportunities are also necessary. This highlights the importance of conceptualizing VR interventions as one component of comprehensive, multi-level approaches to healthcare worker well-being rather than standalone solutions.

### 4.4 Satisfaction

Across studies reporting satisfaction outcomes (15, 16, 21), healthcare workers generally reported positive experiences with VR interventions, expressing satisfaction, perceived helpfulness, and willingness to use them again or recommend them to colleagues. This high acceptability is encouraging for potential implementation, as user engagement and adherence are critical determinants of real-world effectiveness. Several factors may explain this positive response. First, VR’s immersive nature provides novel and engaging experiences that may be more appealing than traditional relaxation techniques (8). Second, the ability to customize virtual environments allows personalization to individual preferences, potentially enhancing relevance and enjoyment (9). Third, VR interventions can be delivered privately and flexibly, addressing concerns about time constraints that may deter healthcare workers from seeking traditional mental health support.

However, satisfaction measures were primarily collected immediately post-intervention using study-specific instruments, limiting comparability across studies and providing no information about whether positive attitudes persist over time. Future research should employ standardized satisfaction measures and assess acceptability at multiple time points to understand factors influencing sustained engagement.

### 4.5 Potential mechanisms of action

Understanding the mechanisms through which VR interventions exert their effects is essential for optimizing intervention design and matching interventions to individual needs. Based on the included studies and broader literature, several mechanisms may be operative: (a) Attentional restoration: According to Attention Restoration Theory, directed attention—the cognitive resource required for focused work—can become depleted with prolonged use, leading to mental fatigue and stress. Exposure to natural environments, even virtual ones, may facilitate restoration of directed attention by engaging involuntary attention and allowing directed attention mechanisms to recover. Several included studies utilized nature-based VR scenarios, supporting this mechanism. (b) Relaxation response induction: VR interventions often incorporate elements designed to elicit relaxation responses, including guided breathing exercises, progressive muscle relaxation, or biofeedback integrated into virtual environments (31). These techniques may directly reduce physiological arousal and subjective stress. (c) Emotional regulation: VR provides safe environments for practicing emotional regulation skills, potentially enhancing coping self-efficacy (32). For healthcare workers exposed to traumatic or stressful events, VR may offer opportunities for emotional processing and debriefing in controlled settings. (d) Distraction and cognitive reframing: Immersive VR experiences may serve as powerful distractions from work-related rumination and worry, interrupting cycles of negative thinking and allowing cognitive reframing. (e) Enhanced engagement and adherence: The novel, immersive nature of VR may increase engagement with and adherence to mental health interventions compared to traditional formats, potentially amplifying and sustaining benefits (26). Future research should explicitly test these mechanistic pathways using mediation analyses and experimental designs that manipulate hypothesized active ingredients.

### 4.6 Limitations

This systematic review has several limitations that should be considered when interpreting findings: (a) Methodological limitations of included studies: A major limitation is that seven of the 10 included studies employed one-group pretest-posttest designs without control groups. This design is particularly susceptible to threats to internal validity, including regression to the mean. These biases may systematically inflate observed treatment effects, leading to overestimation of VR intervention efficacy. Consequently, the pooled effect sizes should be interpreted with caution, as they may reflect not only true intervention effects but also these methodological artifacts. (b) Sample size and statistical power: Several included studies had small sample sizes, limiting statistical power and increasing uncertainty around effect estimates. Small studies may also be more susceptible to publication bias if only those with positive findings are published. (c) Short-term follow-up: The absence of long-term follow-up data in nearly all included studies represents a critical limitation. Current evidence supports only immediate post-intervention effects, and the durability, long-term efficacy, and potential sustained benefits of VR interventions remain entirely unknown. (d) Reliance on self-reported outcomes: All included studies relied exclusively on self-report measures, which are susceptible to response biases, including social desirability bias and expectancy effects. The absence of objective physiological indicators—such as heart rate variability, salivary cortisol levels, or electrodermal activity—limits our ability to corroborate self-reported improvements with objective stress reduction (33). (e) Heterogeneity and publication bias: The high heterogeneity observed, particularly for stress outcomes, reduces confidence in pooled effect size estimates and suggests that effects may vary substantially across contexts and populations. Additionally, due to the limited number of studies included in each meta-analysis (fewer than 10 for all outcomes), we were unable to perform funnel plot analysis or formal statistical tests to assess publication bias, as recommended by the Cochrane Handbook. The possibility of publication bias cannot be ruled out, and unpublished studies with null or negative findings could substantially alter pooled effect estimates. (f) Search limitations: Our search was limited to English and Chinese language publications, and gray literature was not systematically searched, which may have resulted in incomplete inclusion of relevant studies and potential language bias. (g) Inability to meta-analyze key outcomes: The inability to quantitatively synthesize burnout, fatigue, and satisfaction outcomes due to insufficient studies significantly limits the comprehensiveness of our conclusions regarding these important outcomes. Therefore, it is recommended that large-sample, high-quality intervention studies be conducted in future research to explore the optimal VR intervention.

## 5 Conclusion

This systematic review and meta-analysis provides evidence that VR interventions can significantly reduce stress and anxiety among healthcare workers in the short term, with moderate to large effect sizes. Healthcare workers generally report satisfaction with VR interventions and willingness to use them, suggesting good acceptability and potential for real-world implementation. However, these conclusions must be tempered by important limitations: the predominance of uncontrolled study designs, high heterogeneity in stress outcomes, reliance on self-reported measures, absence of long-term follow-up data, and inability to meta-analyze burnout and fatigue outcomes due to insufficient evidence.

The application of VR for healthcare worker mental health remains in its early stages, with substantial research needed to establish efficacy, understand mechanisms, identify optimal intervention parameters, and determine long-term effects. Future research should prioritize rigorous randomized controlled trials with active controls, long-term follow-up, objective outcome measures, and systematic assessment of burnout and fatigue. VR interventions represent a promising but not yet proven approach that warrants continued investigation. With further high-quality research, VR may emerge as a valuable component of comprehensive, multi-level strategies to support the mental health of this essential workforce.
