# Correction: The effect of virtual reality technology on the intervention of mental health problems among healthcare workers: a systematic review

**DOI:** 10.3389/fpubh.2026.1858183

**Published:** 2026-04-29

**Authors:** Tian Chen, Caixia Xie, Xiaoxia Dai

**Affiliations:** 1 School of Medicine, University of Electronic Science and Technology of China, Chengdu, China; 2 Department of Nursing, Sichuan Provincial People's Hospital, University of Electronic Science and Technology of China, Chengdu, China

**Keywords:** anxiety, mental health problem, nurse, physician, stress, virtual reality technology

In the published article, author Caixia Xie was erroneously assigned to affiliation 1 “School of Medicine, University of Electronic Science and Technology of China, Chengdu, China”. This affiliation has now been removed for author Caixia Xie. The affiliation number for the corresponding author has been corrected from “^1, 2^*” to “^2*^” accordingly.

The original version of this article has been updated.

